# Immunomodulatory, behavioral, and nutritional response of tryptophan application on poultry

**DOI:** 10.14202/vetworld.2021.2244-2250

**Published:** 2021-08-28

**Authors:** Nguyen Thuy Linh, Budi Guntoro, Nguyen Hoang Qui

**Affiliations:** 1 Department of Animal Science and Veterinary Medicine, School of Agriculture and Aquaculture, Tra Vinh University, Tra Vinh City, Vietnam; 2 Department of Livestock Social Economics, Faculty of Animal Science, Universitas Gadjah Mada, Yogyakarta, Indonesia

**Keywords:** nutrition, poultry’s diet, poultry’s performance, tryptophan

## Abstract

Tryptophan is an essential amino acid for all animals that was discovered through casein hydrolysis. The use of tryptophan as feed additives has been attracting the attention of many nutritionists because it cannot be synthesized enough in an animal’s body. Tryptophan or precursor to the vitamin niacin in the diet is important, and its supplementation for poultry is determined to improve the amino acid balance and promote the poultry’s growth performance through enhancing appetite, feed efficiency, and protein synthesis. Moreover, poultry in different growth phases, breeding, and conditions require various amounts of tryptophan. In addition, supplemented tryptophan also improves the immune response or the immunomodulatory activity of poultry to various diseases through the kynurenine pathway, especially diseases in the bursa. Furthermore, tryptophan also has a strong relationship with lysine (the ideal tryptophan/lysine ratio) in improving growth performance. However, tryptophan deficiency could affect the behavioral responses (e.g. pecking behavior and poultry stress) because tryptophan serves as a precursor for the neurotransmitter serotonin and the pineal hormone melatonin in the diet. This paper tried to summarize all information about applying tryptophan in the diets and illustrate the roles of tryptophan in the poultry industry.

## Introduction

**Figure-1:**
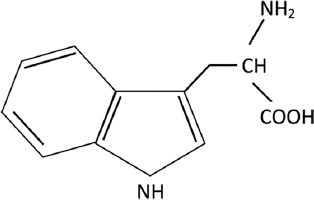
Chemical structure depiction of Tryptophan [4].

The poultry industry has been developing recently to meet the extremely increasing demands of human nutrition. The development of markets has forced the increase and diversification of poultry products in both quality and quantity. Feed formulation is one of the vital methods to increase poultry productivity, and protein in feed plays a crucial role in poultry development. Protein is made up of approximately 20, 10, and 10 amino, essential amino, and nonessential amino acids, respectively [1]. Aside from lysine and methionine, tryptophan was also a limited amino acid that cannot be synthesized enough by the mammal body [2]. In addition, tryptophan is an essential amino acid for monogastric livestock and preweaning ruminants in nutrition [3]. Figure-1 shows the chemical structure of tryptophan[4].

The roles of tryptophan were recorded in previous studies. First, tryptophan helps poultry increase their growth performance through increasing appetite, feed efficiency, and feed utilization [3,5-11]. Second, tryptophan helps poultry synthesize protein for body demand [12]. Third, tryptophan in the diets of poultry records the reduction in stress response and aggressive behavior [13]. Finally, tryptophan has suggested increasing immune response [14] and has strong anti-inflammatory effects [15]. Three common precursors of tryptophan (i.e. niacin, melatonin, and serotonin) were noted. As a niacin precursor, tryptophan in the diet can help poultry decrease fat synthesis in the body. In addition, as serotine and melatonin precursors, tryptophan plays a crucial role in increasing the immune system against various diseases in bursa and functions relating to psychological response [6,16]. Tryptophan serves as a building block for proteins and as a nutrient for nerve function and immune systems and emerges as a critical nutrient for avian nutrition [3].

The aforementioned reasons showed that the study implied to give the overall information of the roles of tryptophan for poultry until currently. Thus, the understanding and knowledge of tryptophan on poultry nutrition are enhanced.

## Sources of Tryptophan

The first report of tryptophan was from Frederick Gowland Hopkins in 1901, who discovered tryptophan by casein hydrolysis. In addition, tryptophan was proved to be essential for animals after mice experimentation [17]. Table-1 shows the tryptophan content in protein and food[18]. Moreover, following the National Center for Biotechnology Information [19], two isomers (i.e. L-tryptophan and D-tryptophan) were noted. L-tryptophan is usually used as a nutritional supplement.

**Table-1:** Tryptophan content of proteins and flours.

Protein sources	n (%)	Tryptophan (g/16 g N)
Proteins		
Casein	13.6	1.70
Lysozyme	16.2	7.66
Soy protein	14.0	1.36
Foods		
Barley flour	1.26	1.55
Beef, minced	13.6	1.25
Corn flour	1.46	1.85
Cottonseed flour	10.0	1.37
Lima bean flour	3.32	1.42
Oat flour	2.56	1.68
Rice flour	0.98	1.72
Soybean flour	8.32	1.43

Source: Friedman [18].

Tryptophan is available in some grains that can be grown in the natural environment. Moreover, tryptophan is known to have a high percentage in nuts and seeds (e.g. cashews, walnuts, peanuts, almonds, sesame, pumpkin, and sunflower seeds) and soybeans [20]. Furthermore, it is available in wheat, rice, and corn. In addition, the production of high-tryptophan transgenic seeds could deal with tryptophan scarcity at a low price because the price of tryptophan is higher than that of normal seeds [18]. The availability of tryptophan will increase if the body consumes such ingredients and then produces more enzymes in the liver and blood [20]. Moreover, tryptophan is available in some common ingredients (Table-2) [21].

**Table-2:** The amount of Tryptophan in common ingredients.

Ingredients	Amount (mg/100 g)
Wheat flour	110
Potato	28
Cheese	325
Banana	10
Soybeans	160
Bread, toasted, and oat bran	140
Chia seed dried	440
Cocoa	290

Source: Kaluzna-Czaplinska *et al.* [21]

**Figure-2:**
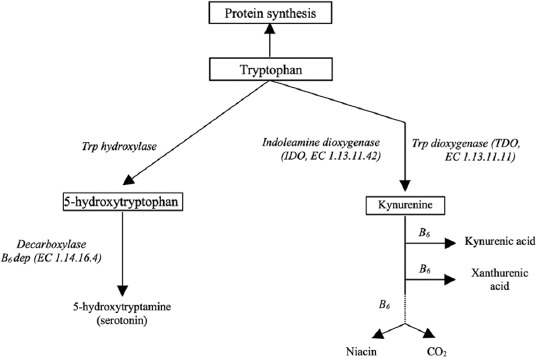
Overview of Tryptophan metabolism [4].

Tryptophan production has three metabolic pathways (Figure-2) [4]: The first (protein), second (serotonin and melatonin), and third (kynurenine) pathways [22]. Like other amino acids, tryptophan contributes to the protein synthesis in the body of animals through the liver. The most important pathway in tryptophan metabolism is in the kynurenine pathway, and the indole molecule plays an important role that is responsible for the production of metabolites with physiological activity in association with body defenses [23]. Moreover, tryptamine, serotonin, and melatonin also bring an indole ring to perform that function to provide antioxidant, anti-inflammatory, and bioactive properties [24-26].

After ingestion, protein is synthesized by ingesting tryptophan in the body [3]. Aside from protein synthesis, the catabolism of tryptophan emerges in the liver through serotonin and kynurenine metabolic pathways. Figure-2 shows that tryptophan is involved in serotonin production that is a neuromodulator, produced in the gut, platelets, and brain of animals. In addition, tryptophan is also metabolized through a specific kynurenine pathway [23]. Tryptophan hydroxylase metabolizes tryptophan into 5-hydroxy tryptophan and further into the serotonin by amino acid decarboxylase and to melatonin through *N*-acetyltransferase [27]. The remaining tryptophan is then degraded through the kynurenine pathway, which is mainly regulated by two rate-limiting enzymes: tryptophan 2,3-dioxygenase and indoleamine 2,3-dioxygenase [27].

## Tryptophan Requirements and its Effect on Poultry

Tryptophan deficiency could lead to many consequences (e.g. a decrease in growth performance and immune system). In animals, tryptophan improves animal’s performances. For example, tryptophan improves growth performance, reduces stress and regulates insulin, synthesizes protein in the muscles, and improves meat quality. For poultry, tryptophan serves as a niacin precursor [28] and melatonin and serotonin precursors [6,16]. Moreover, tryptophan is one of the essential amino acids necessary for growth performance and feed utilization. Poultry could detect tryptophan in the diet and develop an aversion against the tryptophan deficiency in the diet. Feed intake was affected by the amount of tryptophan available in the diet composition. In addition, tryptophan in the diet increases the amount of protein in the body. Furthermore, one of the important records is that tryptophan also increases the economic efficiency of raising poultry [16,29].

## Requirements for Growth Performance

For both body weight gain and feed conversion ratio, 0.17% of tryptophan is required for male broilers. Moreover, 0.17% and 0.16% are required for female broilers for body weight gain and feed conversion ratio, respectively [30]. Chickens supplemented with a suitable amount of tryptophan in the diet gave the highest final live weight, daily weight gain, and greatest feed conversion ratio [5,7-11]. In addition, the availability of tryptophan in the diet has a positive effect on feed intake [31]. Tryptophan is a serotonin precursor [31,32] that manages the appetite of poultry. Furthermore, tryptophan adjusts the appetite and properly utilizes nutrients [32]. The experiment with Cobb 500 chickens recorded significant effects of tryptophan on feed intake and daily weight gain of male broilers as shown in the increase of tryptophan in the diet from 0% to 0.252% [33]. In addition, the best feed conversion was recorded in the treatment of 0.30% tryptophan in the diet compared with other treatments in the experiment [16]. Feed conversion improved to 0.23% of tryptophan in the diet [7] because the amount of tryptophan digested by the poultry increases the protein synthesis in the poultry’s body.

## Requirement for Carcass Characteristics

After measuring blood serum, the supplementation of 0.10% and 0.20% tryptophan in the diet improves total protein, albumin, and glucose for broilers at 49 days old [6]. Similarly, Emadi *et al*. [6] and Preedaa *et al*. [34] showed that the diet decreased the aspartate aminotransferase, triglyceride, and cholesterol, as well as the blood serum, of broilers. Moreover, the supplementation of 0.20%, 0.25%, and 0.30% tryptophan in the diet helps chickens decrease total lipids and total cholesterol in the plasma [16]. Tryptophan in the diet increases not only in serum and liver but also in carcass traits (e.g. breast muscles) [8]. The quadratic effect of tryptophan was noted on carcass yields (e.g. increase in breast meat weight and a decrease in abdominal fat) [33]. In addition, the beneficial effects of tryptophan and protein on morphological features of the small intestine, particularly the greatest length of the jejunum, were recorded in previous studies [35,36]. Moreover, broilers receiving tryptophan in the diet can reduce abdominal fat deposition than those receiving treatment without tryptophan. It may be affected by the amount of tryptophan in the diet that decreases carcass lipid content. Furthermore, tryptophan has beneficial effects on morphological features of the small intestine [36]. The application of tryptophan is reviewed (Table-3) [4,7,10,11,29,30,37-41].

**Table-3:** Tryptophan requirement for classes of poultry.

Classes of poultry	Authors	Tryptophan requirements
Chicks	Abebe and Morris [37]	12 g/kg CP
	Freeman [38]	2.2-2.4 g/kg
	Opoola *et al.* [10]	0.24%
Growing chicks	Duarte *et al.* [7]	0.19-0.22%
	Han *et al.* [39]	0.22%
	Opoola *et al.* [10]	0.21%
	Mund *et al.* [11]	0.3-0.5%
Broiler chicks		
Male broiler	Freeman [38]	0.17%
	Rosa *et al.* [30]	0.17%
Female broiler	Freeman [38]	0.17%
	Rosa *et al.* [30]	0.16-0.17%
Laying pullet	Harms and Russell [4]	149 mg/hen/day
	Calderano *et al.* [41]	142 mg/hen/day
Commercial hens	Russel and Harms [40]	157 mg/hen per day
	Khatun *et al.* [29]	145 mg/hen/day

However, some studies showed that tryptophan did not affect carcass characteristics (e.g. breast meat, thigh, drumstick, back, and wings) [7,11] because tryptophan metabolism is affected not only by the amount of tryptophan in the diet but also by various factors. In addition, tryptophan supplemented in the diet with low protein cannot help chicken compensate for protein deficiency [36].

## Ideal Ratio of Tryptophan and Lysine

Tryptophan has a strong relationship with other amino acids (e.g. lysine) in the diet to increase and improve growth performance. The ideal ratio between essential amino acids (e.g. tryptophan and lysine) was estimated to give the most suitable ratio of these amino acids in the poultry diet. These studies showed that the suboptimal level of digestible lysine in the diet is crucial to determine that all lysine is digestible or not. Thus, accurately estimating the digestible tryptophan/digestible lysine ratio is possible. It is also a way to formulate the amount of tryptophan and other amino acids in the diet.

## Broiler Poultry

In a poultry diet, adjusting the amount of tryptophan/lysine is very important. The ratio of tryptophan/lysine is 19% when using Ross 208 chickens from 20 to 40 days old [42]. The tryptophan diet is suitable for the poultry’s growth between 17% and 19% of lysine in the diet [43]. Particularly, tryptophan/lysine recommended for poultry is 17%, 18%, and 19%, which can help birds improve their body weight gain, feed conversion ratio, and feed consumption. With a lower tryptophan/lysine ratio, 16.6% is recommended as ideal for the poultry’s growth performance [44].

## Laying Poultry

**Figure-3:**
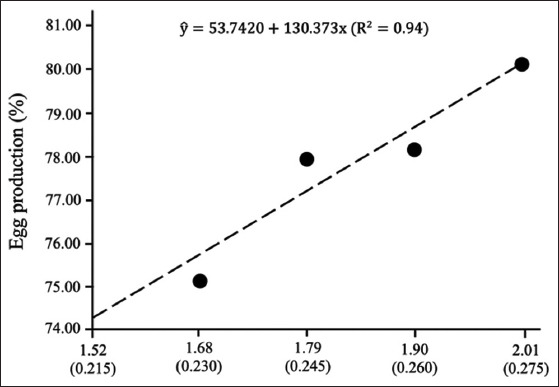
Digestible Tryptophan and Tryptophan: Lysine ratio [8].

Figure-3 showed the correlation between the amount of tryptophan and egg production in laying hens [8]. Moreover, Figure-3 showed that Calderano *et al*. [41] and Lima *et al*. [45] recommended that the ideal ratio of tryptophan and lysine is 25.5% and 24.5%, respectively, which are recommended in the diet for laying hens. Furthermore, 24.3% of tryptophan/lysine is the ideal ratio in the diet [46], whereas 17.5-19% of tryptophan in the diet is recommended as the ideal ratio for laying hens [47].

## Effects of Tryptophan on Poultry Behavior

### Pecking behavior

The previous studies showed that tryptophan is a crucial amino acid in regulating the poultry’s behavior. The poultry is usually in a crowded environment or stressful condition, and these environmental conditions could have strong effects on the behavioral response and generating abnormal social behaviors (e.g. feather pecking behavior) [48]. As an amino acid involved in the synthesis of serotonin and melatonin, tryptophan affects the behavior of poultry through two hormones in the body’s poultry [6]. In addition, the tryptophan–kynurenine pathway plays an important role in feather pecking behavior [49]. Feather pecking behavior is mostly associated with deficiencies in central serotonin during the laying and the rearing periods [50,51]. Moreover, diets without tryptophan could negatively affect the feather pecking behavior of poultry [52]. When an amino acid deficiency in the diet exists, the poultry tries to peck their feather to counteract that deficiency because the feathers have some essential amino acids. Thus, tryptophan supplemented with a suitable level in the diet reduces feather pecking in poultry [53,54].

### Stress response

Tryptophan in the diet affects the stress response of poultry, especially laying hens. Serotonin is known as a central brain neurotransmitter [55]. Moreover, 5-hydroxytryptamine (serotonin) has been shown to regulate aggressive behavior and stress response [56-58]. The behaviors of poultry (e.g. mood, metabolic process, sleep, and growth) are affected by central serotonin [51]. The studies of Crumeyrolle-Arias *et al*. [59] and Yıldırım *et al*. [60] on poultry recorded that stress response and pecking behavior have been affected by the amount of tryptophan in the diet through the indolic pathway. Tryptophan supplementation in the diet may also reduce aggression and diminish stress in many species, including chickens [54]. Moreover, tryptophan deficiency in the diet could affect all kinds of physiological functions [61]. The increase in tryptophan supplementation could lessen oxidative stress and help chickens deal with the stress condition [62]. Moreover, Bai *et al*. [27] showed that tryptophan plays a task in modulating biological functions and reducing stress. Most parameters employed in the method of evaluating the enzymatic antioxidant status are catalase, glutathione reductase, and peroxidase. Meanwhile, the enzymatic and non-enzymatic systems for oxidative stress are represented by total antioxidant status. Moreover, the previous studies on antioxidants using white Pekin ducks have resulted in increased antioxidants and catalase in serum, liver, and chest muscles with dietary tryptophan supplementation [8].

## Effects of Tryptophan on Immune Response

The effects of tryptophan in the diet on the immune system have been investigated in some studies. Through the kynurenine pathway, tryptophan could affect the immune system and have the immunomodulation function [3]. Tryptophan metabolites play a crucial role in various fundamental biological processes (e.g. cell growth and division) or antioxidant status. Yao *et al*. [3] also recorded that tryptophan and its metabolites (e.g. 5-hydroxytryptamine) regulate the function of immunity or play an important role in the immune system response of animals [63]. Strasser *et al*. [20] showed that malignant cells, the attenuation of the growth of infectious agents, and the regulation of the immune system are highly influenced by tryptophan metabolism. This is essential because the immune system status may be drastically influenced by the lower amount of tryptophan in case of continuous activation [20]. The psychological conditions of the animals are impacted in a way that tryptophan affects the livestock immune system [27]. For example, if the livestock is under stress or suffers from inflammation, the tryptophan decomposition is said to help in differentiating the production of lymphocytes and immunoglobulins. Moreover, it also has a positive impact on humoral immunity. Furthermore, lymphocytes and other cells are responsible for the immune responses that usually happen in some organs (e.g. spleen, thymus, and bursa of Fabricius) [8]. Tests conducted on the Yangzhou goose demonstrated that dietary tryptophan improves the spleen index and blood immunoglobulin G (IgG) and immunoglobulin M (IgM) levels [64]. Ducklings also show improvement in the growth of the spleen, bursa of Fabricius, and thymus because of dietary tryptophan. Thus, the immune function and lymphocyte proliferation are reflected by the high spleen index [65].

Moreover, tryptophan has a positive effect on stabilizing nuclear polysaccharides for the synthesis of globulins. However, it causes low levels of plasma IgM and IgG levels when in deficit (Table-4) [11,63]. Infectious bursal disease in chickens is affected by the tryptophan dosage as well [6]. Broiler chicks based on tryptophan 0.3 and 0.5 dietary plan showed better results in glutathione reductase and arylesterase, catalase, glutathione peroxidase, and antioxidant status [11].

**Table-4:** The effect of Tryptophan on immunity of chickens from 7-21 days.

Items	0.3% Tryptophan in the diet	0.5% Tryptophan in the diet
Total antibodies	1.98	2.52
Immunoglobulin G	1.32	1.98
Immunoglobulin M	0.66	0.54

Sources: Mund *et al.* [11]

## Conclusion

Tryptophan plays an indispensable role in poultry performance with the main function of improving the poultry’s performance by enhancing protein synthesis and the immune system. Moreover, the depletion of tryptophan in the diet leads to negative effects on behavioral and immunomodulatory responses of poultry performance.

## Authors’ Contributions

NTL and BG: Designed the concept of the manuscript and collected the information. NHQ: Drafted and corrected the manuscript. All authors read and approved the final manuscript.
